# Visit of Liebig

**Published:** 1850-08

**Authors:** 


LIEBIG, the distinguished chemist, is about to visit the United States, and contemplates giving a series of lectures in the various cities.

